# Focal areas of increased lipid concentration on the coating of microbubbles during short tone-burst ultrasound insonification

**DOI:** 10.1371/journal.pone.0180747

**Published:** 2017-07-07

**Authors:** Klazina Kooiman, Tom van Rooij, Bin Qin, Frits Mastik, Hendrik J. Vos, Michel Versluis, Alexander L. Klibanov, Nico de Jong, Flordeliza S. Villanueva, Xucai Chen

**Affiliations:** 1 Center for Ultrasound Molecular Imaging and Therapeutics, University of Pittsburgh Medical Center, Pittsburgh, Pennsylvania, United States of America; 2 Netherlands Heart Institute, Utrecht, the Netherlands; 3 Department of Biomedical Engineering, Thoraxcenter, Erasmus MC, Rotterdam, the Netherlands; 4 Laboratory of Acoustical Wavefield Imaging, Faculty of Applied Sciences, Delft University of Technology, Delft, the Netherlands; 5 Physics of Fluids Group, MIRA Institute for Biomedical Technology and Technical Medicine and MESA+ Institute for Nanotechnology, University of Twente, Enschede, the Netherlands; 6 Cardiovascular Division, Department of Medicine, University of Virginia, Charlottesville, Virginia, United States of America; Nanjing University, CHINA

## Abstract

Acoustic behavior of lipid-coated microbubbles has been widely studied, which has led to several numerical microbubble dynamics models that incorporate lipid coating behavior, such as buckling and rupture. In this study we investigated the relationship between microbubble acoustic and lipid coating behavior on a nanosecond scale by using fluorescently labeled lipids. It is hypothesized that a local increased concentration of lipids, appearing as a focal area of increased fluorescence intensity (hot spot) in the fluorescence image, is related to buckling and folding of the lipid layer thereby highly influencing the microbubble acoustic behavior. To test this hypothesis, the lipid microbubble coating was fluorescently labeled. The vibration of the microbubble (*n* = 177; 2.3–10.3 μm in diameter) upon insonification at an ultrasound frequency of 0.5 or 1 MHz at 25 or 50 kPa acoustic pressure was recorded with the UPMC Cam, an ultra-high-speed fluorescence camera, operated at ~4–5 million frames per second. During short tone-burst excitation, hot spots on the microbubble coating occurred at relative vibration amplitudes > 0.3 irrespective of frequency and acoustic pressure. Around resonance, the majority of the microbubbles formed hot spots. When the microbubble also deflated acoustically, hot spot formation was likely irreversible. Although compression-only behavior (defined as substantially more microbubble compression than expansion) and subharmonic responses were observed in those microbubbles that formed hot spots, both phenomena were also found in microbubbles that did not form hot spots during insonification. In conclusion, this study reveals hot spot formation of the lipid monolayer in the microbubble’s compression phase. However, our experimental results show that there is no direct relationship between hot spot formation of the lipid coating and microbubble acoustic behaviors such as compression-only and the generation of a subharmonic response. Hence, our hypothesis that hot spots are related to acoustic buckling could not be verified.

## Introduction

Ultrasound contrast agents (UCAs) consist of coated gas microbubbles (1–10 μm in diameter) dispersed in an aqueous suspension. These blood pool agents aid in the diagnosis of for example liver [1] and kidney lesions [2] and in left ventricular visualization [3]. In the blood pool, uncoated microbubbles would dissolve in less than 0.3 s [4] which is too short a lifetime for diagnostic imaging; a coating is therefore essential for increased stability and thus longevity of the microbubbles. The coating reduces the surface tension and the corresponding capillary pressure that drives the gas out of the microbubble core into the surrounding fluid. In addition, it forms a barrier that reduces gas diffusion [5–7].

For medical purposes, ultrasound frequencies ≥ 0.5 MHz are typically used at varying acoustic pressures [8]. When exposed to an ultrasound wave, the gas core of the microbubble responds to the pressure change of the ultrasound by compression and expansion, which results in the vibration of the microbubble [5, 7, 9]. The vibration provides microbubble-specific nonlinear acoustic signals for contrast-enhanced ultrasound imaging (CEUS) [7, 10] and can induce bio effects such as microbubble-mediated drug uptake [5, 9, 11]. In clinically used UCAs, the microbubble coating consists of albumin or lipids; the most prevalent coating consists of lipids [12]. Lipid-coated microbubbles can show various vibration regimes in an ultrasound field, which are characterized by the volumetric vibration dynamics and shape oscillations of the microbubble. These vibrations vary from gentle for imaging and drug uptake applications to violent for drug uptake and cell killing applications [5, 9].

Microbubble behavior has been widely studied to improve CEUS and drug uptake, either by acoustic scattering and attenuation measurements [13–16] or by optical observations using ultra-high-speed cameras [17–22]. The optical ultra-high-speed cameras typically operate in a bright field imaging mode, thereby visualizing the diffract shadow image of the gas core, but not the lipid coating, since the coating cannot be resolved at optical resolution. At large radial excursions the inertial oscillation of the gas core dominates the microbubble vibration, and therefore bright field imaging is sufficient [5]. However, at small radial excursions the behavior of the coating may dominate the vibration of the microbubble [23], which requires a more direct visualization of the coating. For lipid-coated microbubbles, the coating can be visualized by incorporating lipid dyes or fluorescent lipids in the microbubble coating. Borden et al. [24] incorporated the lipid dye DiI in their in-house produced microbubbles which they insonified with repeated one-cycle pulses at a frequency of 2.25 MHz at 400 kPa peak negative pressure (P_). The fluorescence recordings (30 frames per second (fps)) in between the ultrasound pulses revealed bud formation on the lipid coating, characterized by a higher intensity spot of fluorescence signal, as well as formation of lipid strings and globular aggregates, all of which are considered to be a result of the collapse of the lipid monolayer coating [25–27]. These alterations of the lipid coating are likely related to the acoustically-induced microbubble deflation, as Borden et al. [24] measured a decrease in microbubble diameter. Microbubble deflation in the absence of ultrasound at a time scale of seconds has been shown to induce the formation of buckles, folds, and vesicles on the microbubble coating which were visualized by means of the lipid dye DiI [28, 29]. Luan et al. [30] observed bud formation on their in-house produced microbubbles (1 MHz, 255 kPa P_, 500 cycles) after incorporating the lipid dye DiI in the coating and subsequent fluorescence recordings at a frame rate of 150 kfps. This frame rate allowed visualization of bud formation during insonification and also of movements of buds/lipid clusters along the coating interface during insonification. The time scale of the recording, however, was still too low to completely resolve fast the microbubble dynamics.

Although these studies have led to improved understanding of the lipid coating on microbubbles, real-time visualization of the coating during insonification is still lacking. At present it is unknown at which vibration amplitude the lipid monolayer collapses and whether this occurs during the compression or expansion phase. So far, only irreversible collapse of the monolayer has been observed for the microbubble coating [24, 30], while numerical simulations of lipid monolayer dynamics also predict reversible collapse [26]. The relationship between specific microbubble behavior and lipid coating behavior on a molecular scale has also not been studied experimentally before. For example, compression-only behavior, i.e. when a microbubble compresses substantially more than it expands, as observed in optical bright field studies with a fast framing camera [31, 32], can be simulated by the models of Marmottant [33], Doinikov [34], and Paul [35] and is hypothesized to be related to buckling of the lipid coating by the Marmottant model [31]. Buckling of the lipid coating, however, has not yet been verified and observed in optical studies with a fast framing camera. Real-time visualization of the lipid coating on a molecular scale during insonification requires a camera capable of recording fluorescence movies at ultra-high speed (~5 Mfps to record microbubble vibration in a 1 MHz ultrasound field) with high sensitivity and spatial resolution to capture enough signals from fluorophores on a nanoseconds time scale. The recently developed UPMC Cam, an ultra-high-speed camera capable of recording bright field and fluorescence movies at 25 Mfps, meets these requirements [36].

In this study we investigated the relationship between the collapse of the lipid coating of the microbubble and the phenomenon of buckling and compression-only behavior at a tens of nanoseconds time interval. For this purpose, lipid-coated microbubbles were fluorescently labeled by chemically conjugating fluorescent Oregon Green 488 dye to the lipids. We recorded the behavior of the fluorescently labeled microbubble coating while being insonified at an ultrasound frequency of 0.5 and 1 MHz with the UPMC Cam ultra-high-speed camera, operated at ~4–5 Mfps. The behavior of the coating during insonification was then correlated to the acoustic response of the microbubble. It is hypothesized that a local increased concentration of lipids, appearing as a focal area of increased fluorescence intensity (hot spot) in the fluorescence image, is related to acoustic buckling.

## Methods

### Fluorescent microbubbles

Two types of fluorescent microbubbles were fabricated. For type 1 fluorescent microbubbles, lipid-coated microbubbles with a coating of 1,2-distearoyl-*sn*-glycero-3-phosphocholine (DSPC; 2 mg/mL in phosphate buffered saline (PBS); 79 mol%; Sigma-Aldrich, St. Louis, MO, USA) and 1,2-distearoyl-*sn*-glycero-3-phosphoethanolamine-N-[pyridyldithiopropionate (polyethylene glycol)-2000] (DSPE-PEG(2000)-PDP; 2 mg/mL in PBS; 21 mol%; Avanti Polar Lipids, Alabaster, Alabama, USA) were made with a C_4_F_10_ gas core (Fluoromed, Round Rock, Texas, USA) in the presence of 10% (v/v) glycerol (T.J. Baker Chemicals, Avantor Performance Materials, Center Valley, PA, USA) and 20% (v/v) propylene glycol (Sigma-Aldrich) using a Vial Shaker (Lantheus Medical Imaging Inc., N. Billerica, Massachusetts, USA) for 15 s, with 0.8 mL of solution in a 2.5 mL glass vial (Sun SRI, Rockwood, Tennessee, USA). Fluorescent Oregon Green 488-maleimide (ThermoFisher Scientific Inc., Waltham, MA, USA) was then conjugated to the microbubbles. First, 300 μL of 250 mM DL-Dithiothreitol in MilliQ (DTT, Sigma-Aldrich) was added to the microbubbles in the glass vial and the microbubbles were incubated on a Barnstead Thermolyne Labquake Shaker (ThermoFisher Scientific Inc.) at room temperature for 1 h. Then, half of the microbubble/DTT suspension (i.e. 0.6 mL; the other half was discarded) was washed at 400 *g* for 1 min (Beckman Coulter Allegra 25R Centrifuge), and resuspended in 1 mL of 0.8 mg/mL Oregon Green 488 maleimide in C_4_F_10_-saturated PBS. This was followed by an incubation of 2 h on the Barnstead Thermolyne Labquake Shaker at room temperature in the dark. The microbubbles were washed again two times as described and suspended in 1.0 mL of C_4_F_10_-saturated PBS.

Type 2 fluorescent microbubbles were made by coupling succinimidyl ester of Oregon Green 488 carboxylic acid (ThermoFisher Scientific Inc.) to 1,2-distearoyl-sn-glycero-3-phosphoethanolamine-N-[amino(polyethylene glycol)-2000] (DSPE-PEG(2000)-NH_2_; Avanti Polar Lipids). DSPE-PEG2000-NH_2_ reacted with the 20% molar excess of the ester of Oregon Green 488 carboxylic acid in chloroform:DMSO solvent mixture (2:1 volume ratio) in the presence of 20% molar excess of diisopropyl ethylamine base (Sigma-Aldrich). Excess of non-bound dye and base were removed from the conjugate by repeated extractions with aqueous buffered saline, followed by extractions with water, to remove salt. Resulting conjugate was lyophilized for storage and reconstituted in chloroform. Fluorescent microbubbles were made by evaporating the chloroform under Argon gas, drying the lipid film under vacuum for 5 min, and adding DSPC in PBS, glycerol and propylene glycol as described above. The lipid solution in the 2 mL glass vial was sonicated for 5 min in a sonicator bath (20 kHz; Model 75D, VWR International, Radnor, PA, USA) before adding C_4_F_10_. After making the microbubbles in the Vial Shaker, the microbubbles were incubated at room temperature for 1 h in MilliQ, washed, incubated for 2 h in C_4_F_10_-saturated PBS, and washed as for the other microbubbles. All incubation steps after making the microbubbles were thus kept the same.

Microbubble size distributions were measured on a Multisizer 3 Coulter Counter (Beckman Coulter, Indianapolis, IN, USA). A 50-μm aperture tube was used, allowing quantification of microbubble diameters between 0.8 and 18 μm using 300 channels. Measurements were performed in PBS as diluent (20 mL diluent volume; 100 μL analytic volume) and repeated three times to obtain the mean microbubble diameter, size distribution, and concentration.

### Ultra-high-speed fluorescence recordings

After an OptiCell (Nunc, ThermoFisher Scientific; note that this product has been discontinued) was pretreated with 2% bovine serum albumin (Sigma) in PBS for 1 h at room temperature and washed three times with PBS, 5 μL of fluorescent microbubbles were injected in the OptiCell filled with PBS. This resulted in a final concentration of ~1×10^5^ microbubbles/mL in the OptiCell.

During ultra-high-speed imaging, single microbubbles were insonified at room temperature at a frequency of 0.5 MHz (V318-SU, Olympus Scientific Solutions Americas, Waltham, Massachusetts, USA; microbubble type 2) or 1 MHz (A302S-SU-F1.63IN-PTF, Olympus; microbubble type 1 and 2) with a single tapered 10-cycle sine burst unless mentioned otherwise. The ultrasound signal was generated by an arbitrary waveform generator (Agilent 33210A; Keysight Technologies, Santa Rosa, California, USA) and amplified by an amplifier (model 100A250A; Amplifier Research, Souderton, PA, USA). The P_ varied between 25 and 300 kPa, as verified with a 200-μm capsule hydrophone (HGL-0200, Onda Corp, Sunnyvale, CA, USA). Fluorescence microscopic recordings were obtained with the UPMC Cam [36], an ultra-high-speed imaging camera at the University of Pittsburgh Medical Center, equipped with a 60× magnification lens (LUMPLFLN 60X/W, NA 1.0, depth of field 0.61 μm, Olympus Corp, Tokyo, Japan), and pulsed 488 nm laser system (Genesis MX488-5000, Coherent, Santa Clara, CA, USA) driven by a pulsed current power supply (AV-106B-B, Avtech Electrosystems, Ogdensbugh, NY, USA). The 5 W laser source was triggered from the camera system such that the single laser pulse covered all 128 frames, starting 5 μs before the first frame and ending 5 μs after the last frame. The laser beam size covered the whole field of view. Frame rates were ~4–5 Mfps to study the acoustic behavior of the microbubble coating. The frame rate at 0.5 MHz insonification was lower (~4 Mfps) than that at 1.0 MHz insonification (~5 Mfps) to ensure that 10 acoustic cycles at both insonification frequencies were recorded. The resulting spatial resolution of the overall optical system was 0.173 μm/pixel.

### Analysis

Microbubbles were initially included in the study if they had not been insonified previously and if they were ‘single’ microbubbles, i.e. the neighboring microbubbles were at least two microbubble diameters away. Recordings of microbubbles were excluded if experimental errors had occurred, such as the microbubble was out of focus, or if the recording did not cover the full 10 cycles of ultrasound.

Custom-designed image analysis software [37] was used to obtain *D–t* curves from all fluorescence microbubble recordings. The original software was designed for bright field images and tracked the inner inflection point of the fluorescent coating, underestimating the size of the microbubble. By inverting the minimum cost algorithm of the analysis software, the outer inflection point of the fluorescent coating could also be tracked. The mean between the inner and outer contour of the coating was then taken to obtain the size of the fluorescence microbubble during the recording. To improve the sensitivity, the *D*-*t* curves were resampled digitally at a 10 times higher sampling rate, resulting in 1280 points per curve instead of the original 128 data points.

The initial microbubble diameter, *D*_0_, was determined from the average signal before the microbubble started to vibrate. The average diameter after the ultrasound burst was used as the final diameter, *D*_end_. The ratio between the *D*_end_ and *D*_0_ was used to quantify the diameter change after a single ultrasound burst.

The *D*-*t* curves derived from the ultra-high-speed fluorescence movies were analyzed for the degree of microbubble expansion and compression. The maximum diameter (*D*_max_) and the minimum diameter (*D*_min_) were determined from the part of the *D*-*t* curve during which the microbubble was vibrating. The oversampling enabled a more accurate determination of *D*_max_ and *D*_min_, which would otherwise only be based on a few data points and therefore sensitive for outliers. Symmetric behavior was defined elsewhere [31] for *E*/*C* = 0.5–2, compression-only behavior for *E*/*C* < 0.5, and expansion-only behavior for *E*/*C* > 2, where *E* = (*D*_max_−*D*_0_)/ *D*_0_ is the relative expansion, and *C* = (*D*_0_ –*D*_min_)/ *D*_0_ is the relative compression. An additional parameter that was calculated based on *E* and *C* is the relative vibration amplitude, defined as (*E*+*C*)/2.

The *D*-*t* curves were transformed to the frequency domain using a Fast Fourier Transformation (FFT), since analysis in this domain provides specific information on the frequency content of the recorded signal. The maximum vibration amplitude in a frequency band of 300 kHz centered around the transmit frequency (*f*_T_) was determined. Likewise, the maximum amplitudes of the FFT were determined in a 300 kHz frequency band centered at $\frac{1}{2}f_{T}$ for the subharmonic frequency, 2*f*_T_ for the second harmonic frequency, and 3*f*_T_ for the third harmonic frequency (the sampling rate was sufficiently high to determine 3*f*_T_ only for the 0.5 MHz insonification) [19]. When the amplitude at these frequencies was at least 6 dB above the noise level, these microbubbles were classified as responsive at the respective harmonic frequency [19]. The noise level was estimated from the average of the FFTs of microbubble recordings without the application of ultrasound (*n* = 38 for 0.5 MHz; *n* = 18 for 1 MHz).

As a more robust measure for the asymmetry of a *D*-*t* curve we used the method introduced by Sijl et al. [20]. First, the *D*_0_ was subtracted from the original signal and the FFT was recalculated. Next, the low frequency component *A*_0_ was extracted from the time signal, expressing the offset of the *D*-*t* curve, using a low-pass Butterworth filter with a cut-off frequency at 125 kHz for *f*_T_ = 0.5 MHz and at 250 kHz for *f*_T_ = 1 MHz. The maximum negative amplitude of *A*_0_ is a measure for the compression and the uncertainty in the determination was estimated from the maximum of *A*_0_.

Before, during, and after insonification, the presence or formation of hot spots, defined as a focal area of increased fluorescence intensity, was determined manually in each recording.

### Statistics

The diameter change results were expressed in a Tukey box and whisker plot. Comparisons were performed using a one-way ANOVA in GraphPad InStat version 5.04 (GraphPad Software). Differences were considered significant if *p* < 0.05.

## Results

**Fig 1:**
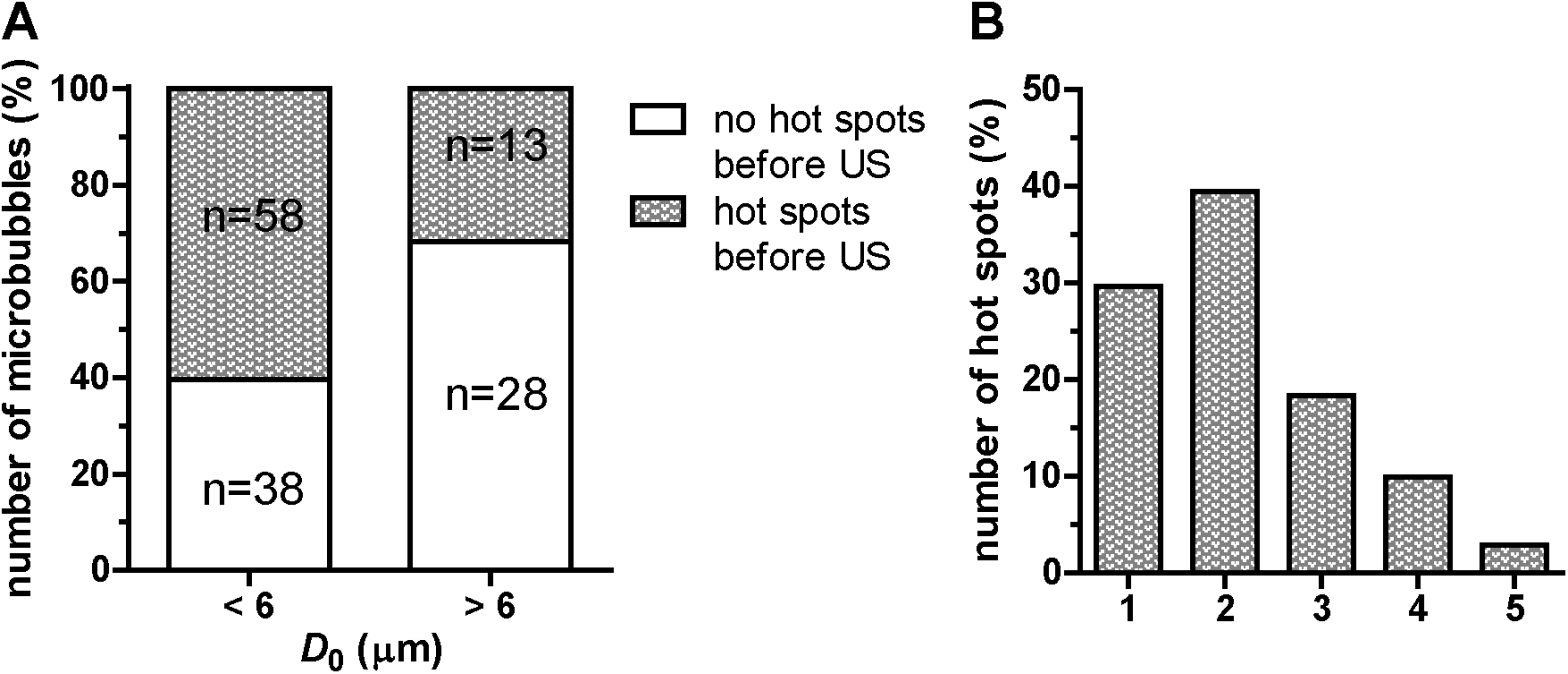
Homogeneity of the fluorescent microbubble coating before insonification. (A) Number of microbubbles with hot spots (*n* = 137 microbubbles). (B) Number of hot spots per microbubble coating (*n* = 71 microbubbles). Hot spots were defined as focal areas of increased fluorescence intensity.

**Fig 2:**
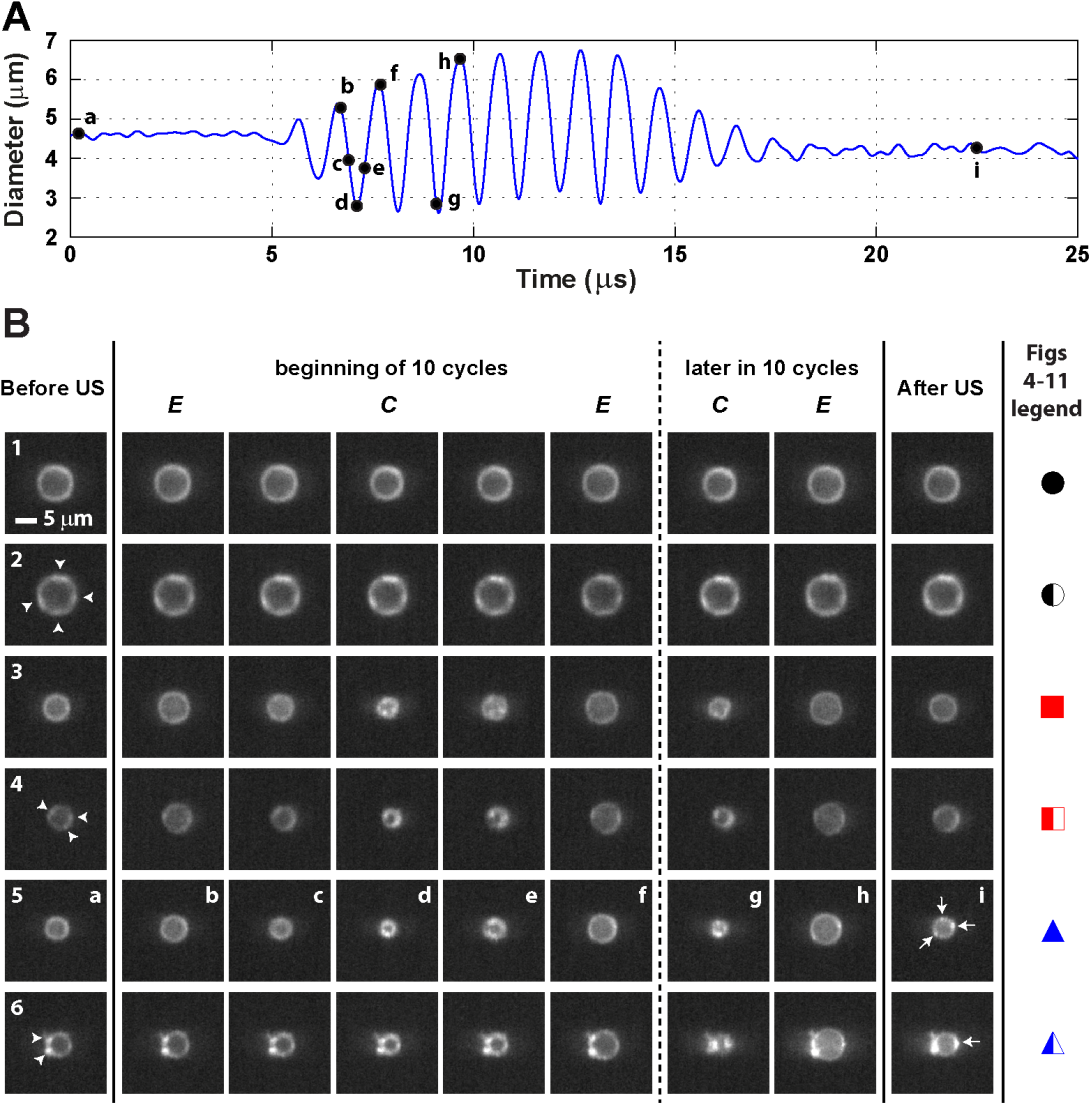
Microbubble vibration during insonification at a frequency of 1 MHz and P_ of 50 kPa. (A) *D*-*t* curve of the microbubble shown in 2B5; the letters correspond to the frames shown in 2B5. (B) Typical examples of lipid coating behavior during insonification. Arrow heads in leftmost column indicate hot spots present before insonification; arrows in second column to right indicate hot spots formed during insonification that persisted when the ultrasound was off; see S1–S6 Videos for the ultra-high-speed recordings of the shown selected frames. US = ultrasound; *E* = expansion phase; *C* = compression phase. The symbols in the rightmost column (see Fig 3 for more details) are the figure legends for Figs 4–10.

The number weighted mean microbubble diameter as determined from the Coulter Counter measurements was 3.7 μm with a standard deviation of diameter of 2.6 μm. In total, 137 randomly selected microbubbles that met the inclusion and exclusion criteria were studied optically, where the smallest microbubble had a diameter of 2.3 μm and the largest microbubble was 10.3 μm in diameter. About half of the microbubbles (51.8%; *n* = 71) showed inhomogeneities in the fluorescent coating before insonification. These were defined as focal areas of increased fluorescence; hereafter referred to as “hot spots”. Interestingly, the occurrence of hot spots was higher in smaller microbubbles (*D*_0_ < 6 μm) as shown in Fig 1A. On average 2.2 hot spots per microbubble coating were observed, with a range of one to five as shown in Fig 1B. Typical examples of these hot spots before ultrasound application are given in Fig 2B2, 2B4, and 2B6 (leftmost column) for three different microbubbles. The microbubble in Fig 2B2 had four hot spots, the one in Fig 2B4 three, and the microbubble in Fig 2B6 had two hot spots, all indicated by arrow heads.

**Fig 3:**
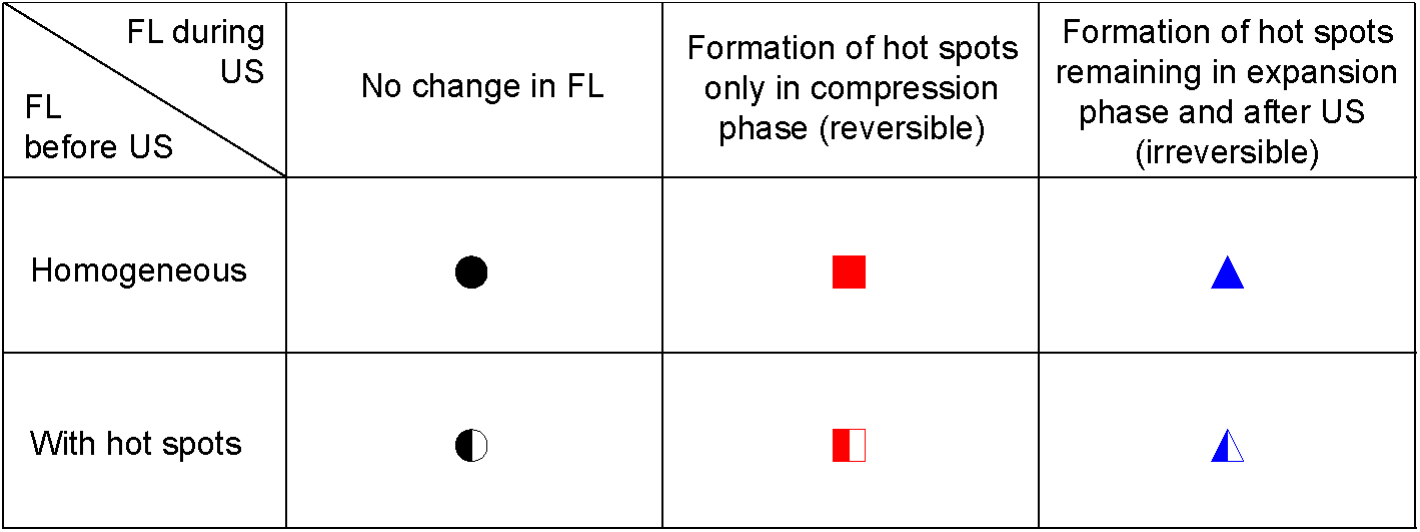
Categories of fluorescence observations during insonification. The symbols in the Fig are also used as Fig legend for Figs 4–11; see Fig 2B. FL = fluorescence; US = ultrasound.

When the microbubbles were insonified at a frequency of 0.5 MHz and at a P_ of 25 or 50 kPa or at a frequency of 1 MHz and at a P_ of 50 kPa, we either observed no change in fluorescence (Fig 2B1 and S1 Video and Fig 2B2 and S2 Video) or the formation of new hot spots within the first acoustic cycle, i.e. on a microsecond scale (Fig 2B3–2B6 and S3–S6 Videos), irrespective of whether microbubbles had hot spots before ultrasound application. The hot spots present before insonification remained visible in the compression and expansion phase as shown in Fig 2B2, 2B4 and 2B6. The new hot spots always appeared first during the compression phase of the microbubble vibration, and in 68% of the cases these new hot spots also persisted in the expansion phase and after the ultrasound was turned off, suggesting the formation of reversible (Fig 2B3 and 2B4) or irreversible (Fig 2B5 and 2B6) hot spots within the coating. For the microbubble in Fig 2B5 three new irreversible hot spots were formed, while the microbubble in Fig 2B6 only formed one, indicated by arrows in the second to right column of Fig 2. These newly formed irreversible hot spots were more pronounced at the second half of the 10-cycle sine burst and persisted in the expansion phase of the microbubble. On average 1.9 new hot spots per microbubble coating (range 1–6, see Table 1) were formed during insonification, irrespective whether they were reversible or irreversible, and an average of 1.7 new hot spots (range 1–6, see Table 1) persisted when the ultrasound was turned off. Overall, the fluorescence behavior was divided into six categories, depending on the presence of either a homogenous distribution of fluorescence or hot spots in the microbubble monolayer coating before ultrasound, and whether and when, these hot spots were observed during insonification, as summarized in Fig 3. The symbols in Fig 3 are also depicted in the rightmost column of Fig 2 to illustrate which symbol correlates to which observed fluorescence behavior.

**Table 1:** Amount of newly formed hot spots on the coating of microbubbles.

Newly formed hot spots	Amount of hot spots per microbubble coating (%)					
	1	2	3	4	5	6
**Reversible (during US)**	**48**	**22**	**22**	**9**	**0**	**0**
**Irreversible** **(during US)**	**50**	**29**	**4**	**13**	**2**	**2**
**Irreversible (persisting after US is off)**	**58**	**31**	**4**	**2**	**2**	**2**

US = ultrasound.

**Fig 4:**
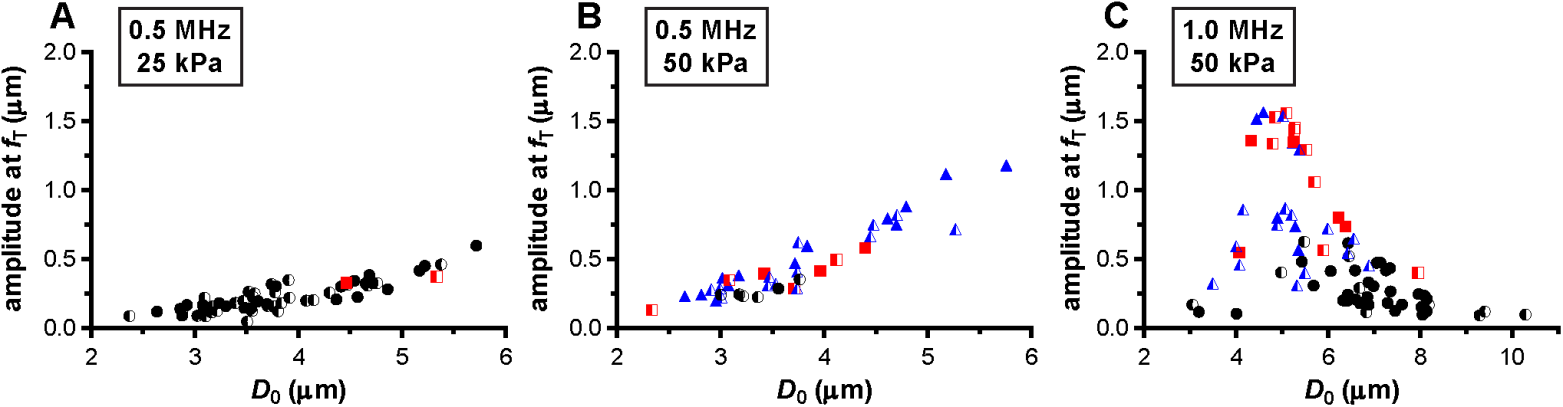
Amplitude at the fundamental frequency, *f*_T_. For microbubbles insonified at a frequency of 0.5 MHz and a P_ of 25 kPa (A), frequency of 0.5 MHz and P_ 50 kPa (B), and frequency of 1 MHz and P_ of 50 kPa (C). For figure legend, see the rightmost column of Fig 2 and Fig 3.

**Fig 5:**
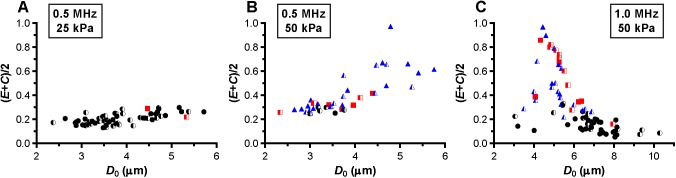
Relative vibration amplitude, (*E*+*C*)/2. For microbubbles insonified at a frequency of 0.5 MHz and a P_ of 25 kPa (A), frequency of 0.5 MHz and P_ of 50 kPa (B), and frequency of 1 MHz and P_ of 50 kPa (C). For figure legend, see the rightmost column of Fig 2 and Fig 3.

**Fig 6:**
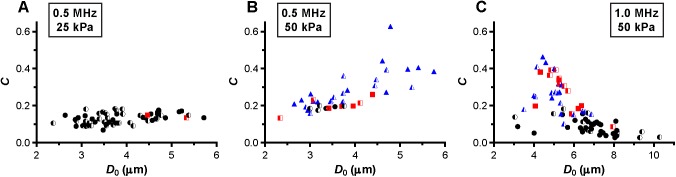
Relative compression amplitude, *C*. For microbubbles insonified at a frequency of 0.5 MHz and P_ of 25 kPa (A), frequency of 0.5 MHz and P_ of 50 kPa (B), and frequency of 1 MHz and P_ of 50 kPa (C). For figure legend, see the rightmost column of Fig 2 and Fig 3.

At a frequency of 0.5 MHz, the microbubbles were insonified below their resonance frequency as indicated by the increase in the maximum vibration amplitude at the transmit frequency, *f*_T_, for larger microbubbles (Fig 4A and 4B). At 25 kPa, only two out of the 60 microbubbles (3%) showed reversible hot spots during insonification (red squares in Fig 4A); the other 58 microbubbles showed no change in fluorescence (97%, black spheres in Fig 4A). The half open symbols indicate microbubbles that already had hot spots before insonification. When these microbubbles were insonified again at 0.5 MHz and 50 kPa (Fig 4B), only 6 out of 40 microbubbles (15%) showed no change in fluorescence. The other microbubbles (85%) either formed reversible or irreversible (blue triangles in Fig 4) hot spots during insonification. At a frequency of 1 MHz and 50 kPa (Fig 4C), the fundamental amplitude was maximal for microbubbles with a *D*_0_ of 4.5–5 μm, indicating these microbubbles were insonified near their resonance frequency. Around resonance, the majority of the microbubbles formed hot spots, while most of those above resonance showed no change in fluorescence. Microbubbles insonified at their resonance frequency vibrate at their largest vibration amplitude, and the resonance size of the microbubble type used in our study was identified from Fig 5C and found to be 4.5–5 μm. The microbubbles insonified at a frequency of 0.5 MHz and a P_ of 50 kPa (Fig 5B) had a larger relative vibration amplitude than those insonified at a frequency of 0.5 MHz and a P_ of 25 kPa (Fig 5A). Above a relative vibration amplitude of 0.3, all microbubbles formed hot spots, irrespective of the insonification frequency. Likewise, above a relative compression amplitude of 0.2, all microbubbles formed hot spots (see Fig 6). No clear distinction between reversible and irreversible hot spots could be observed, also not in the relative expansion amplitude of the microbubbles.

**Fig 7:**
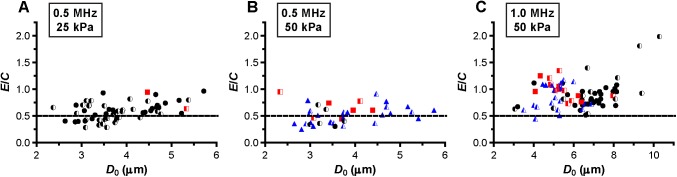
E/C. For microbubbles insonified at a frequency of 0.5 MHz and P_ of 25 kPa (A), frequency of 0.5 MHz and P_ of 50 kPa (B), and frequency of 1 MHz and P_ of 50 kPa (C). For figure legend, see the rightmost column of Fig 2 and Fig 3. Microbubbles with an *E*/*C* < 0.5 show compression-only behavior, indicated by the dashed line.

**Fig 8:**
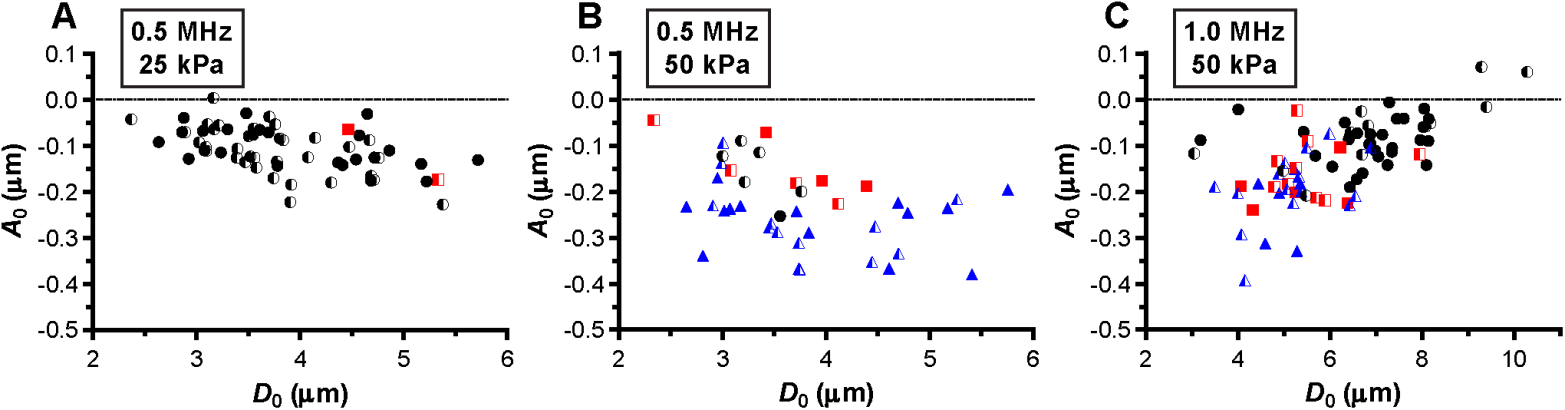
The maximum negative amplitude of *A*_0_. For microbubbles insonified at a frequency of 0.5 MHz and P_ of 25 kPa (A), frequency of 0.5 MHz and P_ of 50 kPa (B), and frequency of 1 MHz and P_ of 50 kPa (C). For figure legend, see the rightmost column of Fig 2 and Fig 3.

Next, we assessed whether a relationship between hot spots and compression-only behavior was present. Several microbubbles showed compression-only behavior, indicated by *E*/*C* < 0.5, predominantly when insonified at a frequency of 0.5 MHz (Fig 7A and 7B). Those microbubbles either had no change in fluorescence (mainly at a frequency of 0.5 MHz and P_ of 25 kPa; Fig 7A) or formed reversible or irreversible hot spots. The formation of hot spots in the coating also occurred when microbubbles vibrated symmetrically, *E*/*C* ~1, most profoundly for 1 MHz insonification frequency and P_ of 50 kPa (Fig 7C). The maximum negative amplitude of *A*_0_ is another measure for the compression of the microbubble and we observed that microbubbles were more likely to form hot spots for larger *A*_0_ (Fig 8).

**Fig 9:**
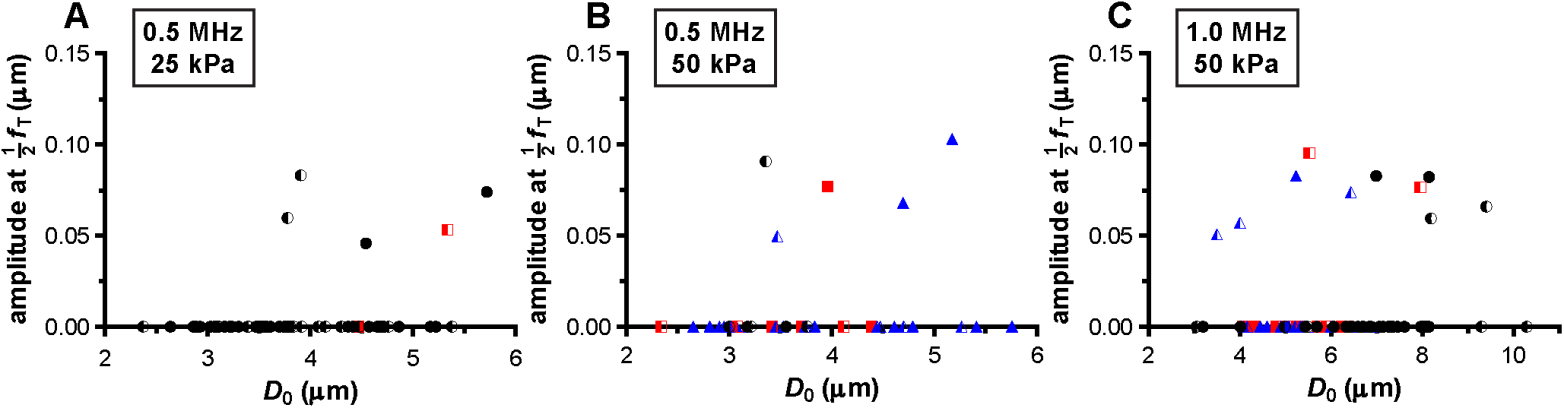
Amplitude at the subharmonic frequency, $\frac{1}{2}f_{T}$. For microbubbles insonified at a frequency of 0.5 MHz and P_ of 25 kPa (A), frequency of 0.5 MHz and P_ of 50 kPa (B), and frequency of 1 MHz and P_ of 50 kPa (C). For figure legend, see the rightmost column of Fig 2 and Fig 3. A value of 0 indicates no subharmonic was present.

**Fig 10:**
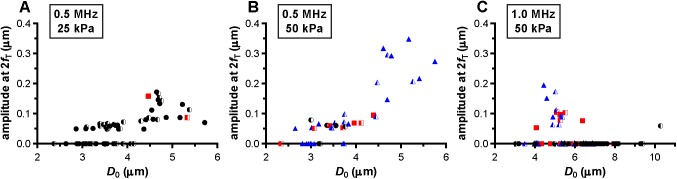
Amplitude at the second harmonic frequency, 2*f*_T_. For microbubbles insonified at a frequency of 0.5 MHz and P_ of 25 kPa (A), frequency of 0.5 MHz and P_ of 50 kPa (B), and frequency of 1 MHz and P_ of 50 kPa (C). For figure legend, see the rightmost column of Fig 2 and Fig 3. A value of 0 indicates no second harmonic was present.

We also assessed whether a relationship between hot spots and the response at the harmonic frequencies was present. At a frequency of 0.5 MHz and P_ of 25 or 50 kPa, five of the microbubbles (8% at P_ of 25 kPa; 13% at P_ of 50 kPa) had a measurable response at the subharmonic frequency; at a P_ of 25 kPa one out of these five (20%) formed a hot spot while this was four out of five (80%) at a P_ of 50 kPa (Fig 9). From the 77 microbubbles insonified at 1 MHz and 50 kPa, the response at the subharmonic frequency was present in ten microbubbles (13%); six of these (60%) formed a hot spot (Fig 9C). No differences were observed between the presence of a response at the subharmonic frequency and microbubbles that did or did not have hot spots before insonification. A response at the second harmonic frequency was present in the majority of the microbubbles that were insonified at a frequency of 0.5 MHz (64% at 25 kPa and 73% at 50 kPa). At a frequency of 1 MHz and a P_ of 50 kPa, 12% of the microbubbles had a response at the second harmonic frequency. All three types of lipid coating behavior, *i*.*e*. no change in fluorescence, formation of reversible, and irreversible hot spots, were amongst the microbubbles that had a response at the second harmonic frequency, see Fig 10. For the microbubbles insonified at 0.5 MHz, we could also assess the presence of a response at the third harmonic frequency. Three out of the 60 microbubbles (5%) insonified at 25 kPa had a response at the third harmonic frequency; none showed a change in fluorescence. At 50 kPa, 16 out of the 40 microbubbles (40%) had a response at the third harmonic frequency which contained a mix of all three types of lipid coating behavior.

**Fig 11:**
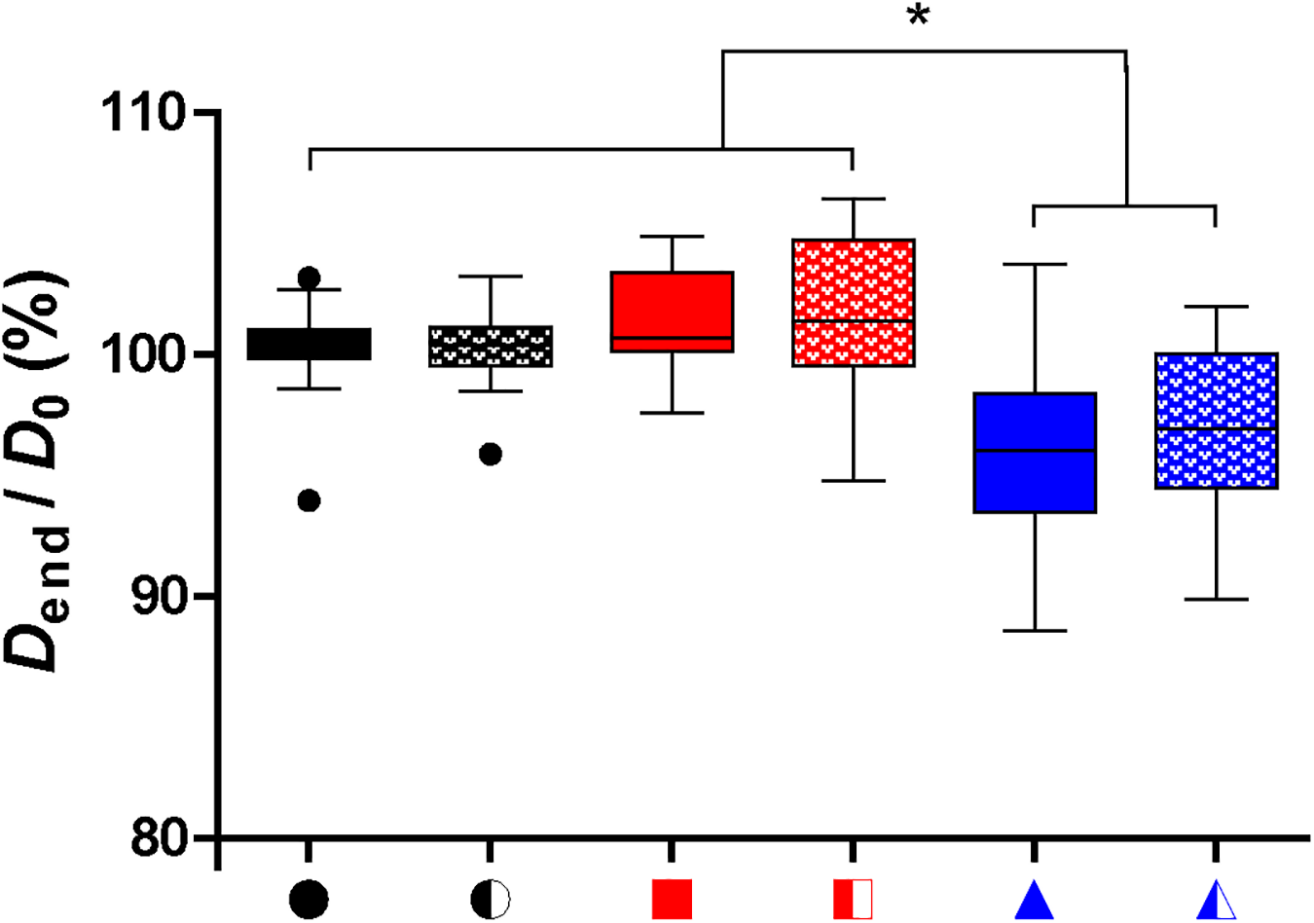
Relative microbubble diameter after insonification. Combination of microbubbles insonified at a frequency of 0.5 MHz and P_ of 25 and 50 kPa and frequency of 1 MHz and P_ of 50 kPa; * indicates significant difference; for the symbols on the x-axis, see the rightmost column of Fig 2 and Fig 3.

Fig 11 shows the change in the diameter of the microbubble between *D*_0_ and *D*_*end*_, the final diameter after insonification. Microbubbles that had formed an irreversible hot spot were significantly smaller than microbubbles that showed no change in fluorescence or that formed a reversible hot spot. This was irrespective of insonification frequency and applied P_.

**Fig 12:**
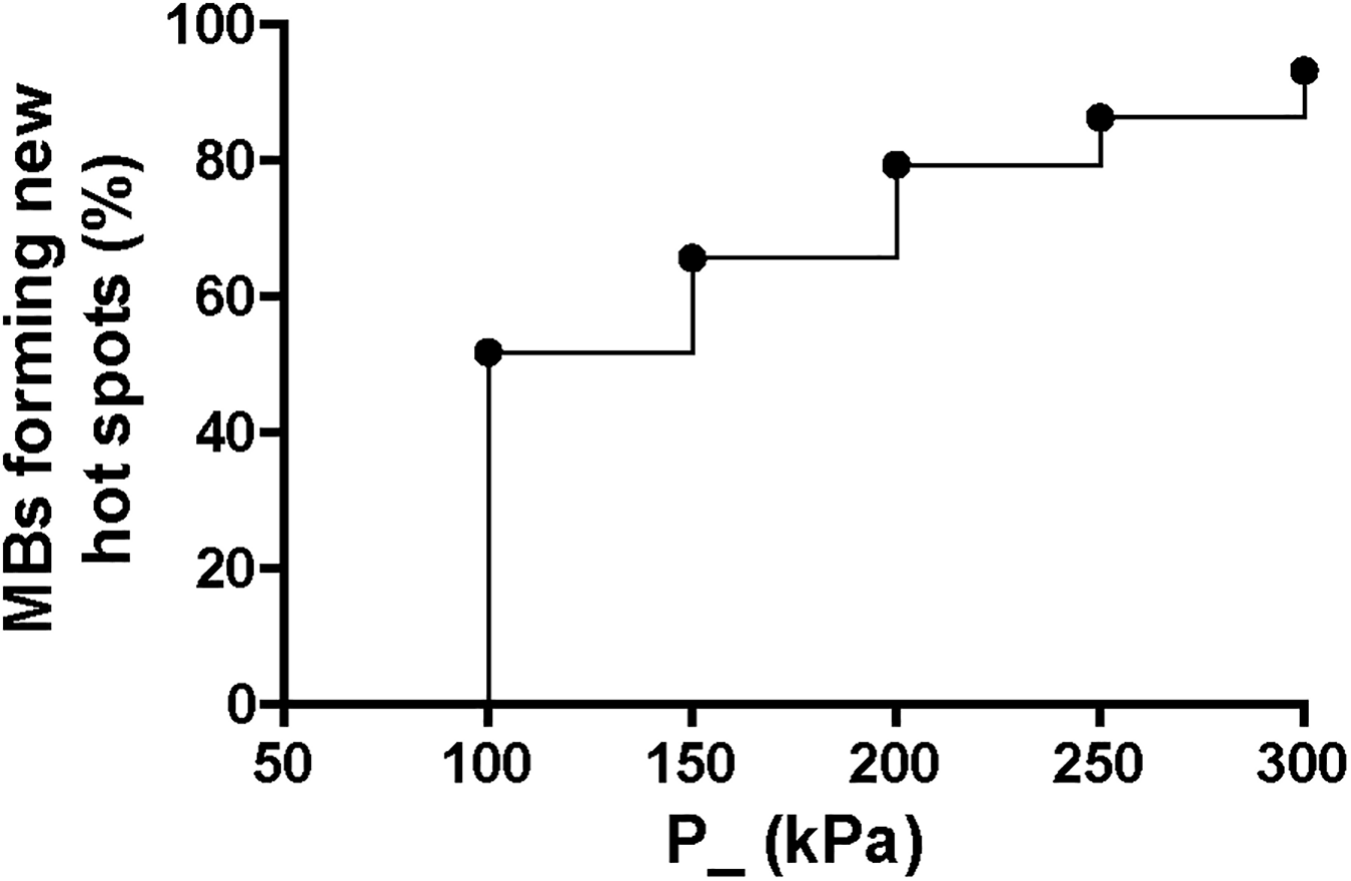
Percentage of microbubbles forming hot spots at frequency of 1 MHz and higher P_. *n* = 41 microbubbles.

**Fig 13:**
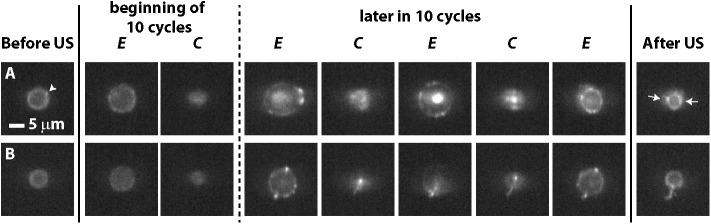
Microbubbles insonified at frequency of 1 MHz and higher P_. Insonification at P_ of 300 kPa and 10 cycles (A) or P_ of 150 kPa and 15 cycles (B). Arrow head in leftmost column indicates hot spot present before insonification; arrows in rightmost column indicate hot spots formed during insonification that persisted when the ultrasound was off; see S7 and S8 Videos for the ultra-high-speed recordings of the shown selected frames. US = ultrasound; *E* = expansion phase; *C* = compression phase.

On a subset of microbubbles that showed no change in fluorescence at a frequency of 1 MHz and P_ of 50 kPa (*n* = 41), the P_ was increased to 300 kPa in steps of 50 kPa. As shown in Fig 12, the majority of microbubbles formed hot spots at higher P_, *i*.*e*. at higher relative vibration amplitudes. For 12 out of these 41 microbubbles (30%), the formation of hot spots could not be determined because insonification was not assessed for all higher P_ (*n* = 7), or due to technical errors (*n* = 5). An example of a microbubble insonified at 300 kPa is shown in Fig 13A. The microbubble had one hot spot before insonification, indicated by the arrow head in the leftmost column of Fig 13A. In the expansion phases, a non-continuous lipid coating was observed, most evident during expansion later in the 10 cycles, suggesting that the coating had ruptured. In that same expansion phase, a bright fluorescence intensity spot was observed in the center of the microbubble. This signal could originate from a smaller microbubble that has temporarily pinched off from the original microbubble in the lateral direction, or a jetting phenomenon, as both observed before with bright-field ultra-high-speed imaging in side view [38]. After insonification, two more hot spots were present indicated by the arrows in the rightmost column of Fig 13A. The diameter of this microbubble was 30% smaller after insonification. For one microbubble insonified at a P_ of 150 kPa and 15 cycles, we observed the formation of a 5 μm long ligment in fluorescence signal (Fig 13B) that was not evident in bright field. This microbubble had a 10% smaller diameter after insonification.

## Discussion

To the best of our knowledge, this is the first study that investigates on a nanoseconds time scale the dynamic lipid motion in the microbubble coating during insonification. We observed three different types of behavior of the fluorescently labeled lipid coating: (a) no change in fluorescence; (b) reversible hot spot formation during insonification (only in compression phase); (c) irreversible hot spot formation during insonification (in compression and expansion phase that persisted after ultrasound was turned off). Hot spots were first formed in the compression phase when the relative vibration of the microbubble was > 0.3, irrespective of the insonification frequency (0.5 or 1 MHz) and P_ (25 or 50 kPa).

### Hot spots

Although observations of ultrasound-induced hot spot formation on the microbubble coating were made in earlier work by others [24, 30], our new ultra-high-speed recordings show that these hot spots form on a nanosecond time scale. In our study, the formation of hot spots on the microbubble coating was first observed in the compression phase of the microbubble vibration. From lipid monolayer studies it is known that lipids condense upon lateral compression. If further compression is applied, the 2D lipid monolayer collapses into a 3D structure. Upon further compression, the following 3D structures can be formed: buckles, bilayer folds, vesicles, tubes, or micelles [25–27]. Although the resolution of our microscopic systems does not allow us to observe the structure of the hot spots in detail, the hot spots are likely to be the result of lipid monolayer collapse through buckles, folds or vesicles than microstructures, although it is known that DSPC-based microbubbles have microstructures in their coating [28, 39–41]. Others have shown that folds in fluorescently labeled lipid monolayer films have a higher fluorescence intensity than microstructures [42, 43] irrespective of the type of lipids in the monolayer and the fluorescent dye. In addition, folds and vesicles at the interface of DSPC-based microbubble coatings were also observed by Owen et al. [44] and Kim et al. [41] for their in-house produced microbubbles, albeit with electron microscopy (coating composition DSPC:PEG-40 stearate in molar ratio 9:1 and 10:1, respectively). Folds and vesicles were also observed on deflated in-house produced microbubbles by Longo et al. [28] (coating composition phosphatidylcholines in various acyl chain lengths including DSPC: DSPE-PEG(2000) in molar ration 9:1) and by Pu et al. [29] (coating composition phosphatidylcholines in various acyl chain lengths including DSPC:PEG-40 stearate in molar ratio 10:1) by fluorescence microscopy.

An average amount of 1.9 reversible or reversible new hot spots were observed on the microbubble coating during insonification. The difference between reversible and irreversible lipid monolayer collapse is governed by the molecular composition of the monolayer and temperature, *i*.*e*. the monolayers’ morphology and material properties [25–27]. DSPC, the main lipid (79 mol%) in our microbubble coating formulation, is always in the condensed phase while DSPE-PEG2000 can be in the expanded or condensed phase depending on the surface pressure [45]. Lipids in a condensed phase are semi-crystalline and therefore too brittle to bend, so upon compression a formed bilayer fold breaks at the point of attachment to the monolayer and deposits an independent fragment on top of the monolayer. This results in irreversible collapse because the collapsed materials cannot reincorporate into the monolayer when the surface pressure decreases [25, 27]. This suggests that the reversible or irreversible collapse of microbubbles depends on the initial phase of the lipids and thus on the initial surface pressure/lipid density of the microbubble coating. It may also explain why the microbubbles that formed irreversible hot spots have deflated, since it is likely that gas can more easily escape through fractures in a monolayer, and/or the increased surface tension leads to higher gas pressure and thus smaller gas volume. Although different lipid densities and surface pressures of the microbubble coating are predicted [20, 23] and incorporated in for example the Marmottant [33] and Paul [35] models for microbubble behavior, future experimental studies are needed to verify this.

In our study, 50.4% of the microbubbles already had hot spots before insonification. Our observation that significantly more microbubbles with diameters < 6 μm had hot spots than microbubbles > 6 μm, may suggest that the microbubbles < 6 μm originated from larger microbubbles that deflated after preparation. Microbubble deflation following preparation has been postulated [46] and reported when microbubbles are produced using flow-focusing techniques [47–49]. The deflation is driven by the Laplace overpressure inside the microbubble due to a difference in surface tension at the gas-surrounding fluid interface. The pressure-driven gas diffusion stabilizes when the internal and external gas pressures equalize, which occurs when the lipid domains reform and reach a packing density that eliminates the surface tension and resulting Laplace capillary pressure [46, 49, 50]. As mentioned before, microbubble deflation may result in the formation of buckles, folds, and vesicles on the microbubble coating [28, 29], which is in line with the higher number of hot spots we observed for the smaller microbubbles (*D*_0_ < 6 μm).

### Resonance of microbubbles

The in-house produced microbubbles with a coating of DSPC (79 mol%) and DSPE-PEG(2000) (21 mol%) insonified at a frequency of 1 MHz and a P_ of 50 kPa were resonant for a diameter of ~4.5–5 μm (Fig 4C). This resonance size is lower than what has been reported for other in-house produced microbubbles with a coating of DSPC (59.4 mol%), polyethyleneglycol-40-stearate (PEG-40 stearate) (35.7 mol%), and DSPE-PEG(2000) (4.9 mol%) at 1 MHz, namely ~7.5 μm in diameter (50 kPa insonification) [51]. For another type of DSPC-containing microbubble, 10 μm diameter BR14 microbubbles were resonant at 1 MHz (< 40 kPa insonification) [37]. Although it is known that several factors have an influence on the resonance frequency, such as the applied P_ [15, 52] and composition of the lipid coating [19], our finding that smaller microbubbles were at resonance at 1 MHz could also be explained by the difference in diameter as measured with fluorescence and bright field. Microbubbles appear larger in bright field than in fluorescence, because complex images are obtained in bright field due to refraction, *i*.*e*. diffraction, and scattering of the incident light, while the fluorescence signal directly originates from the fluorescent molecule in the microbubble coating [53].

### Acoustic behavior of microbubble in relation to hot spots

In our study, a relative vibration > 0.3 induced the formation of reversible or irreversible hot spots on the coating of our in-house produced DSPC-based microbubbles. Luan et al. [30] also found a relative vibration threshold of 0.3 for a different lipid behavior of their DPPC-based microbubble coating namely shedding of lipids, defined by the detachment of lipid material from the coating and subsequent transport of the shed material away from the microbubble. Possible explanations why Luan et al. [30] observed shedding and we did not at the identical 1 MHz insonification frequency could be the higher P_ (50–255 kPa vs 50 kPa in our study), longer burst length (100 vs 10 cycles in our study), different microbubble coating (DPPC:DSPE-PEG(2000) in molar ratio 65:35 versus DSPC:DSPE-PEG(2000) in molar ratio 79:21 in our study), or the choice of dye (DiI not chemically linked to the lipids vs Oregon Green 488 covalently linked to the DSPE-PEG(2000) lipid in our study). The influence of the type of microbubble coating is further supported by the findings of Borden et al. [24] who reported that changing the type of lipid coating resulted in differences in the mechanism of lipid shedding for microbubbles that acoustically deflated.

Luan et al. [30] reported that shedding occurred at the observed relative vibration of 0.3 indicating an approximated ~50% surface reduction of the microbubble during the compression phase [30], which is close to the 41% surface reduction postulated for the collapse of a microbubble lipid monolayer [54]. Assuming that the microbubbles vibrated spherically, collapse of the microbubble lipid monolayer as assessed in our study by the formation of hot spots, was observed for a 55% median surface area reduction during the compression phase (interquartile range 47–75%; *n* = 71, *i*.*e*. all microbubbles that formed hot spots in compression), which is indeed close to the postulated 41%.

De Jong et al. [31] hypothesized that microbubble shell buckling is related to compression-only behavior, defined as *E*/*C* < 0.5. In addition, it is generally thought that buckling highly influences the vibration of the microbubble in terms of compression-only and response at the subharmonic frequency [20, 31, 33, 55, 56]. At the start of our study, it was hypothesized that a locally increased concentration of lipids, appearing as a focal area of increased fluorescence intensity (hot spot) in the fluorescence image, is related to buckling and thus influences the microbubble vibration. However, this hypothesis could not be experimentally confirmed because none of the acoustic behavior of the microbubble that we observed related to the formed hot spots. For instance, hot spot formation was observed for microbubbles with *E*/*C* < 0.5, but hot spots were also formed for symmetrically vibrating microbubbles (*E*/*C* ~ 1.0). On the other hand, there were also many microbubbles, especially at a frequency of 0.5 MHz and a P_ of 25 kPa, that had an *E*/*C* < 0.5 without forming a hot spot. Our findings do suggest that the relation between compression-only behavior and hot spot formation only exists when the relative microbubble vibration > 0.3. In line with previous observations that compression-only is more pronounced in smaller microbubbles [20, 31], we also found that compression-only behavior was most present in microbubbles < 4 μm (Fig 6). Whereas *E* and *C* are based on two points in the *D*-*t* curve, *D*_max_ and *D*_min_, respectively, *A*_0_ is determined on the trend of the whole 10-cycle sine burst in the *D*-*t* curve. Although there is a relationship between A_0_ and A_1_ and thus E/C, no threshold value for compression-only behavior based on A_0_ has been determined before. We did observe most irreversible hot spot formation when *A*_0_ was < -0.2 (Fig 7); we therefore suggest -0.2 as the threshold for irreversible hot spot formation.

Subharmonic responses are theoretically related to non-linear behavior of coated and non-coated microbubbles [57]. On the basis thereof, experimentally derived acoustic responses of microbubbles at the subharmonic frequency have been theoretically linked to compression-only behavior [58], but only experimentally observed in a few BR14 microbubbles using ultra-high-speed imaging by Sijl et al. [32, 55]. On the contrary, van Rooij et al. [19] observed subharmonic responses only in the *absence* of compression-only behavior (defined as *E*/*C* < 0.5) in DSPC and DPPC-based microbubbles, in similar conditions as our current. In the present study, 20 out of the 177 microbubbles (11%) had a response at the subharmonic frequency. Only two out of those 20 microbubbles also had an *E*/*C* < 0.5. Both of these microbubbles were insonified at 0.5 MHz, but at different pressures. The microbubble insonified at P_ of 25 kPa had an *A*_0_ of -0.13 (with *E*/*C* = 0.43; no change in fluorescence during insonification) while this was -0.27 for the microbubble insonified at P_ of 50 kPa (with *E*/*C* = 0.38; formation of irreversible hot spot during insonification). We also did not observe a clear relationship between the presence of a subharmonic response and a hot spot prior to insonification, since 60% of the microbubbles already had a hot spot before insonification. In addition, a similar amount of subharmonic responses was detected in microbubbles that did (45%) or did not show hot spot formation during insonification (55%, the sum of 20% hot spots only in compression and 35% hot spots in compression that persisted in expansion and after the ultrasound was off). Therefore, our study did not show any evidence of a relation between subharmonic response and hot spots.

### Experimental considerations

To record lipid microbubble coating behavior on a nanosecond scale during insonification, we used the UPMC Cam and recorded the behavior in top view. A feature of this camera is that it records in 2D, which does not allow for discrimination between the different types of monolayer collapse (*i*.*e*., a buckle, fold, vesicle *etc*.) or the extension of the hot spot out of the focal plane. Simulations [26] have shown that the pathway of monolayer collapse from buckling to folding to vesicle formation is independent of monolayer composition, compression method, and compression rate, so assessing monolayer collapse by hot spot formation in 2D is already sufficient. On the other hand, lateral non-spherical microbubble shapes could be influenced by hot spots prior to insonification or have an influence on the formation of hot spots during insonification, which can be observed with side view recordings [38]. Because of the use of 2D recordings, the observed formed hot spots could have moved out of the focal plane during vibration. Consequently, they may appear to be no longer present. This has been observed by Luan et al. [30] for longer pulses, but only at a framerate of 150 kfps. On the one hand this may explain why we observed reversible and irreversible hot spots. On the other hand, hot spots present before insonification remained visible during the microbubble vibration (see Fig 2B2, 2B4 and 2B6) which suggests that movement or rotation of hot spots out of the recording plane is negligible for short tone-burst insonification.

A microbubble with a diameter of 4 μm is expected to have 20.9×10^6^ molecules of Oregon Green 488 dye on its coating. This number is based on previous work where a number of 2×10^6^ lipids per μm^2^ were reported for microbubbles [59]. The additional mass of the Oregon Green 488 dye (molecular weight (MW) 463 g/mol, Thermo Scientific Inc.) on the acoustic behavior of the microbubble can be neglected. First, Oregon Green 488 is only 14% of the total mass of the DSPE-PEG(2000)-Oregon Green 488 lipid (MW 3,343 g/mol). Second, the effective mass of the microbubble harmonic oscillator is 4πρ_w_R^3^, where ρ_w_ is the density of water (998 kg/m^3^), which for a 4 μm microbubble is 1×10^−13^ kg, i.e. more than 1000 times the mass of the ~2 nm shell. Moreover, we have reported in another study that the addition of streptavidin, which has a mass 130 times larger than Oregon Green 488, to the DSPE-PEG(2000)-biotin lipid does not change the resonance frequency of DSPC-microbubbles [60].

### Implications

Although we observed focal areas of increased fluorescence intensity (hot spots), hypothesized to be buckles as a result of a local increase in phospholipid concentration, we could not relate these to acoustic behavior of the microbubble such as compression-only and subharmonic responses as others did experimentally or theoretically [32, 55, 58]. Because we could not relate the presence of hot spots to the acoustic behavior of the microbubble, this could imply that the hot spots we observed were not buckles. However, we found significant evidence to assume the hot spots are in fact buckles or folds on a nanometer or sub micrometer scale. On the other hand, in the microbubble field the term ‘buckling’ has always been associated with bright field visualization of a larger part of the microbubble (macroscopic) and has been modelled as such. Therefore the definition of buckling may need refinement in terms of the formation of localized buckles on one hand, and the appearance of global buckling which affects the acoustic behavior on the other hand.

Our ultra-high-speed fluorescence observations of the dynamic behavior of the lipid monolayer on coated microbubbles during insonification can be extrapolated to microbubble vibration studies in which the lipid monolayer coating cannot be visualized. If the microbubble has a relative vibration > 0.3, the lipid monolayer will collapse. Combined with acoustic deflation of the microbubble, the monolayer collapse is likely to be irreversible. Our findings may also hold for other lipid coatings and insonification frequencies, but this requires experimental verification.

## Conclusion

Using ultra-high-speed fluorescence recordings, we observed the formation of focal areas of increased fluorescence or hot spots, on the lipid monolayer microbubble coating at relative vibrations > 0.3 at a frequency of 0.5 and 1 MHz at a P_ of 25 and 50 kPa. Around resonance, the majority of the microbubbles formed hot spots. Formation of hot spots was always observed in the compression phase and in 68% of the cases they also persisted in the expansion phase and after the ultrasound was turned off. If the microbubble also acoustically deflated, hot spot formation was likely irreversible. While we have observed that acoustic vibration leads to the formation of hot spots, we did not find a correlation of hot spot formation with nonlinear acoustic behavior of the microbubble. Therefore, we could not verify the previous hypothesis that monolayer collapse by buckling or folding of the lipid coating of the microbubble on a molecular scale leads to nonlinear acoustic behavior of the microbubble.

## Supporting information

S1 Video Ultra-high-speed fluorescence recording of microbubble shown in Fig 2B1. No change in fluorescence during insonification at 1 MHz and P_ of 50 kPa (10 cycles). The video is 34.6 by 34.6 μm and is played at 12 fps.
(AVI)

S2 Video Ultra-high-speed fluorescence recording of microbubble shown in Fig 2B2. The microbubble had four hot spots prior to insonification. No change in fluorescence during insonification at 1 MHz and P_ of 50 kPa (10 cycles). The video is 34.6 by 34.6 μm and is played at 12 fps.
(AVI)

S3 Video Ultra-high-speed fluorescence recording of microbubble shown in Fig 2B3. Formation of hot spots only in compression phase (reversible) during insonification at 1 MHz and P_ of 50 kPa (10 cycles). The video is 34.6 by 34.6 μm and is played at 12 fps.
(AVI)

S4 Video Ultra-high-speed fluorescence recording of microbubble shown in Fig 2B4. The microbubble had three hot spots prior to insonification. Formation of hot spots only in compression phase (reversible) during insonification at 1 MHz and P_ of 50 kPa (10 cycles). The video is 34.6 by 34.6 μm and is played at 12 fps.
(AVI)

S5 Video Ultra-high-speed fluorescence recording of microbubble shown in Fig 2B5. Formation of hot spots in compression phase and remaining in expansion phase during insonification at 1 MHz and P_ of 50 kPa (10 cycles) and after ultrasound is off (irreversible). The video is 34.6 by 34.6 μm and is played at 12 fps.
(AVI)

S6 Video Ultra-high-speed fluorescence recording of microbubble shown in Fig 2B6. The microbubble had two hot spots prior to insonification. Formation of hot spots in compression phase and remaining in expansion phase during insonification at 1 MHz and P_ of 50 kPa (10 cycles) and after ultrasound is off (irreversible). The video is 34.6 by 34.6 μm and is played at 12 fps.
(AVI)

S7 Video Ultra-high-speed fluorescence recording of microbubble shown in Fig 13A. The microbubble had one hot spot prior to insonification at 1 MHz and P_ of 300 kPa (10 cycles). The video is 34.6 by 34.6 μm and is played at 12 fps.
(AVI)

S8 Video Ultra-high-speed fluorescence recording of microbubble shown in Fig 13B. Insonification at 1 MHz and P_ of 150 kPa (15 cycles). The video is 34.6 by 34.6 μm and is played at 12 fps.
(AVI)
